# Time-lapse imaging of *Drosophila* testis for monitoring actin dynamics and sperm release

**DOI:** 10.1016/j.xpro.2021.101020

**Published:** 2021-12-15

**Authors:** Tushna Kapoor, Pankaj Dubey, Krishanu Ray

**Affiliations:** 1 Department of Biological Sciences, Tata Institute of Fundamental Research, Mumbai, Maharashtra 400005, India

**Keywords:** Cell Biology, Developmental biology, Microscopy, Model Organisms

## Abstract

Here we describe a simple step-by-step protocol for collecting high-resolution, time-lapse images of intact *Drosophila* testis *ex vivo* for a limited period using a confocal microscope, with minimum photo-toxic damage, to monitor spermatid individualization, coiling, and release. The F-actin dynamics during spermatid morphogenesis can be further investigated through laser ablations, Fluorescence-Recovery-After-Photobleaching, and drug treatments, using this protocol.

For complete details on the use and execution of this protocol, please refer to Dubey et al. (2016), Dubey et al. (2019), and Kapoor et al. (2021).

## Highlights

• Protocol for time-lapse imaging of *Drosophila* testis ex vivo
• The protocol is useful for monitoring spermatid maturation and release
• Also, for high-resolution analysis of subcellular F-actin dynamics

## Graphical abstract

**Figure:**
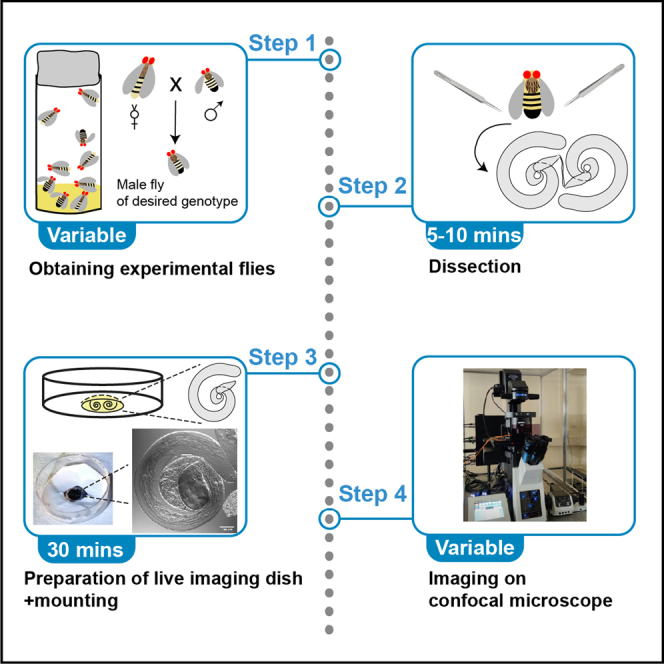

## Before you begin

F-actin dynamics is essential for cellular and tissue morphogenesis, as it forms the underlying basis for various cellular processes such as shaping the plasma membrane, cell migration, cytokinesis, endocytosis, etc. (Blanchoin et al., 2014)(Skau and Waterman, 2015). Actin and plasma membrane remodelling are closely interlinked, and the causal relationship between them is an active area of recent investigation in cell biology (Kessels and Qualmann, 2021). The process of spermatid maturation and release in *Drosophila* testis provides an excellent opportunity to study the molecular cell biology of the interplay between F-actin dynamics and membrane sculpting at the tissue and organ level *ex vivo*. For example, the indentation of the head cyst cell membrane by mature spermatids was shown to induce two different forms of F-actin assembly at distinct time scales using time-lapse confocal imaging from isolated *Drosophila* testis (Dubey et al., 2016)(Kapoor et al., 2021). This system provided an opportunity to observe subcellular changes in the F-actin dynamics and correlate them with the morphogenetic processes in different genetic backgrounds and in the presence of specific function-blocking reagents, using an inexpensive and straightforward protocol.

This protocol specifically describes how to collect 5D (x, y, z, t, and multi-channel) images of F-actin structures associated with the maturing spermatid heads in *Drosophila* testis at high resolution using a confocal microscope, with minimal equipment and sample preparation. This technique has been standardized using simple 1**×** Phosphate Buffered Saline (PBS) as the culture medium. This improvisation allows the isolated testis to be imaged for an extended period of time (4–6 h) on the confocal microscope, with minimal peristalsis of the tissue. Previously used imaging techniques require the use of an insect culture medium with nutrient supplements (Sheng and Matunis, 2011)(Nelson et al., 2020), which leads to a high rate of peristalsis of the testis muscle layer, making it challenging to monitor rapid actin dynamics. The technique described by Noguchi and Miller (2003) requires the isolation of the cyst from the testis. Thus, the imaging was not carried out in the native tissue conditions and should be considered as *in vitro* imaging in a culture medium (Noguchi and Miller, 2003). We tried imaging in several media conditions to minimize the testicular movement, *viz.* 1) Schneider’s insect medium supplemented with 10% non-heat inactivated Fetal Bovine Serum and 1 μg/mL bovine pancreatic insulin (SCM), 2) Schneider’s insect medium (SIM) without Serum and insulin, 3) *Drosophila* medium M1, 4) Jan and Jan buffer and 5) 1**×** PBS. The testicular muscle movement was highest in the SCM and was least in PBS. Hence, we decided to use PBS for our *ex vivo* imaging of *Drosophila* testis. The technique described here allows for simple, non-invasive, reproducible, long-term, and highly adaptive imaging of *Drosophila* testis *ex vivo*. This protocol allowed us to monitor actin dynamics and maintained the coiling and subsequent release of spermatids from the testis into the seminal vesicle for up to 6 h after dissection.

**Figure 1:**
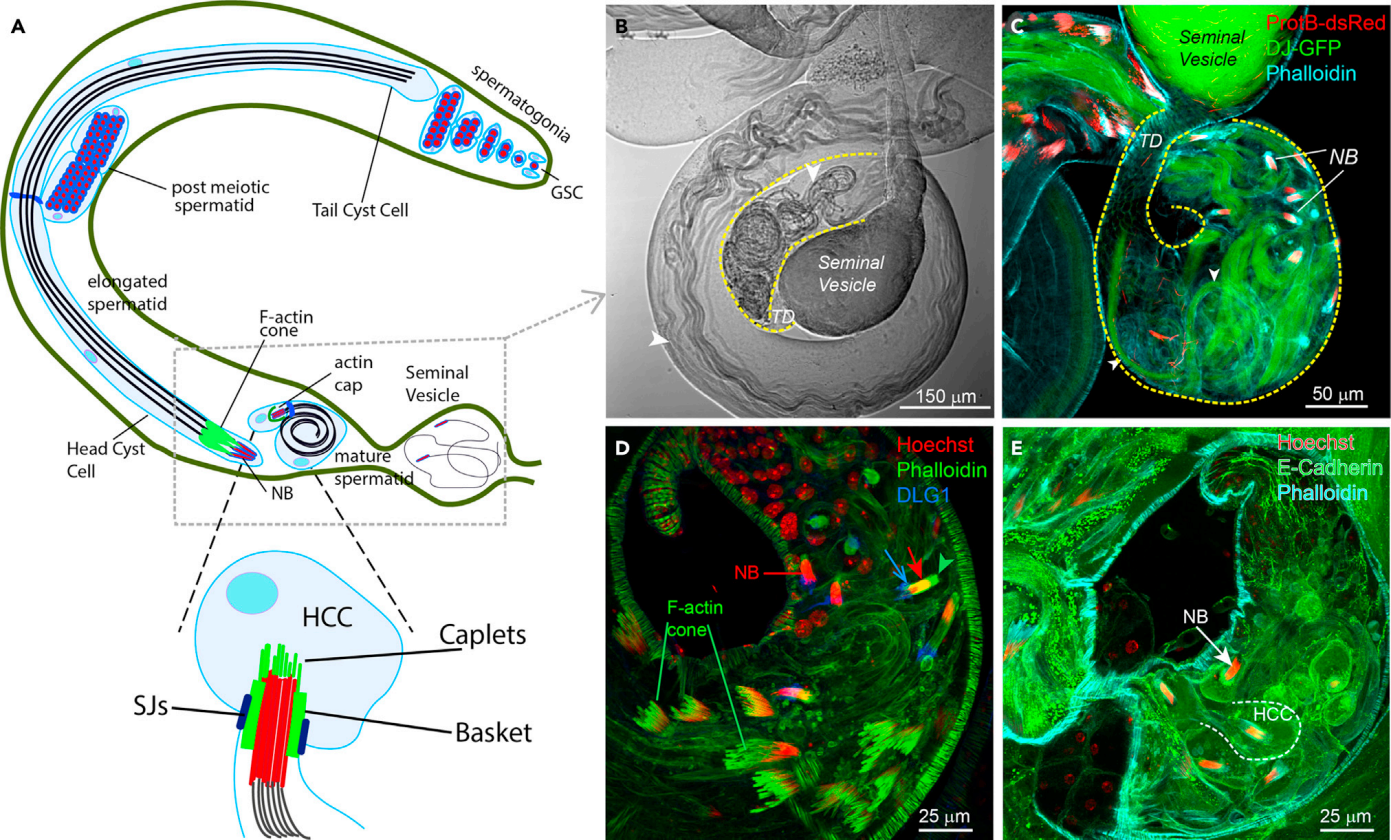
Organization of spermatid head bundles at the base of the adult testis (A) Schematic illustrates the progression of spermatogenesis, from the germline stem cell (GSC) at the testis apex to the mature sperm in the seminal vesicle at the base of the testis. Images of the boxed region, which is also the region of interest for time-lapse imaging of spermatid maturation and release, are shown in panels B-E. (Glossary: NB – nuclei bundle, HCC – head cyst cell, SJs – septate junctions). (B) DIC image of the testis base indicating coiled spermatid tails (arrowheads) in the terminal epithelium (TE) region marked by the yellow dashed line. TD indicates the testicular duct connecting the testis base to the seminal vesicle. (C) The TE zone of a *dj-GFP/protB-dsRed* testis stained with TRITC conjugated Phalloidin. DJ-GFP marks the spermatid tails (arrowheads), and ProtB-dsRed marks the mature spermatid head bundles (NBs). Phalloidin staining indicates the actin caps around mature NBs in the TE region (for details, see (Dubey et al., 2016) (D) The TE zone of a testis from the dlg1-GFP protein trap adult expressing Dlg1-GFP fusion protein under the endogenous promoter stained with the Hoechst dye (red) and TRITC-Phalloidin (green). The red arrow indicates NB, the green arrowhead indicates the actin cap, and the blue open arrow indicates SJs marked by DLG1 (for detail, see Dubey et al., 2019). (E) The TE zone of a testis from the ECadh-GFP protein trap adult expressing E-cadherin-GFP fusion protein under the endogenous promoter stained with the Hoechst dye (red) and FITC-Phalloidin (cyan). The arrow indicates NB, and the head cyst cell (HCC) boundary is indicated by a white dashed line (for detail, see Kapoor et al., 2021)

We have used this technique to image various stages of the sperm maturation and release process, including individualization, coiling, actin cap dynamics, and sperm release (Figure 1). We primarily used stocks expressing fluorescent biomarkers of F-actin and post-meiotic spermatid heads. Here, we describe the procedure using a transgenic fly stock expressing the actin-binding domain of Utrophin fused to GFP, expressed under the spaghetti squash promoter (*sqh::utr-GFP*) and the DNA binding protein ProtamineB fused to dsRed (*ProtB-dsRed*), expressed under its native promoter. The Protamine complex replaces Histones during spermatid maturation and labels the mature spermatid heads. Using this protocol, we have also imaged the morphogenesis of septate junctions (SJs) using neuregulin-GFP protein trap transgenic line (*Nrg-GFP*), which marks the SJs between the cyst cells (Dubey et al., 2019), and the myosin motor localization and dynamics at the actin cap using the GFP-tagged myosin light chain (*squash-GFP*) transgene expressed under its own promoter (*sqh::sqh-GFP*) (Kapoor et al., 2021). The same protocol is used to describe sperm release (Dubey et al., 2016) and the spermatid tail coiling (this study) using DonJuan-GFP (*DJ-GFP*), which marks the mitochondrial derivatives along the sperm tail. The protocol can also be used to collect images through wide-field microscopy and under different culture conditions, and it can be adapted for imaging events in isolated testis from other insects.

### Preparing experimental flies

**Timing: 2–3 weeks**

These steps describe the procedure for generating experimental male flies expressing the F-actin marker Utr-GFP with fluorescently marked mature spermatid heads (genotype - *sqh::utr-GFP/+; ProtB-dsRed/ +*) through a simple one generation cross as described below. Any other relevant genotypes can be similarly generated. In case the experiment demands imaging only actin dynamics, one must cross the homozygous *sqh::utr-GFP* flies to wild-type control, such as the *Canton S* or *Oregon R*, and use the F1 males of the genotype *sqh::utr-GFP/+* for the experiment. Double copies of *sqh::utr-GFP* under homozygous conditions alter actin dynamics.

The flies are grown on standard cornmeal media.

1. Preparation of *Drosophila* growth medium: Mix Cornflour (83 g/mL), dextrose (50 g/mL), agar powder (12 g/mL), and yeast tablet (15 g/mL) in sterile water. Boil the mixture for 10 min while mixing with a stirrer; allow it to cool to 60°C; then add propionic acid (4% v/v), orthophosphoric acid (0.06% v/v), methyl-4-hydroxy benzene (0.07% v/v), while stirring. Pour ∼25 mL in each vial. Allow it to solidify and cool.
2. Set the cross of *sqh::utr-GFP* and *ProtB-dsRed* flies using a 2:1 ratio of young virgin females and males. Put a minimum of 10–12 flies per vial and maintain the vials at 25°C.
3. Every three days, transfer the flies to fresh media, and store all vials at 25°C. Care should be taken to prevent yeast overgrowth in the vials as this affects the spermatid maturation process. Check all the vials daily, and add a few drops of distilled water if the media appeared dry during the larval stages.
4. As the progeny emerge, collect the male flies every few hours, and incubate them at 25°C for 2–4 days.
5. Flip the vials daily to prevent yeast overgrowth.

**CRITICAL:** Do not use vials that contain overgrowth of yeast or fungus as the progeny will not be healthy, and the sperm bundles will appear disrupted.

### Preparation of testis imaging media and drug reconstitution, if applicable

**Timing: 20 min**

6. Prepare 10**×** Phosphate Buffered Saline (PBS) according to (Sambrook and Russell, 2001). Ensure the pH is set to 7.4
7. Dilute to 1**×** using distilled water. Recheck the pH and reset to 7.4, if required.

***Optional:*Reconstitution of pharmaceutical reagents** – The small molecule reagents are reconstituted in DMSO or distilled water, as per the manufacturer’s instructions. The drugs used for perturbing actin dynamics and their bath concentrations are as follows: Rockout (Rho kinase inhibitor) at 200 μM; SMIFH2 (Formin inhibitor) at 25 μM; CK666 (Arp2/3 inhibitor) at 250 μM; Latrnculin A and B (inhibits F-actin barbed end growth) at 5 and 10 μM, respectively; Taxol (microtubule-stabilizing drug) at 10 μM; Colchicine (inhibitor of microtubule polymerization/microtubule-depolymerizing agent) at 10 μM; Cytochalasin D (inhibitor of actin polymerization) at 20 μM; and Jasplakinolide (F-actin stabilizing drug) at 50 μM. Aliquot the drug solutions, and follow manufacturers’ instructions for storage.

**Pause point:** The PBS and drug can be stored for up to a few months. Check manufacturers’ instructions for the storage period of drugs.

### Preparation of petri-dishes for live imaging

**Timing: Overnight (∼16 h)**

This procedure involves drilling a hole at the bottom of a 35 mm petri-dish and fixing a coverslip below the hole. One can either buy 35 mm glass-bottom Petri plates or prepare the chamber as described here (see Methods video S1 for a demonstration).

8. Drill a round hole of diameter 8 mm through the bottom of a 35 mm plastic Petri dish using a motorized drill.
9. Prepare a 9:4 (w/w) mixture of myristic acid wax and petroleum jelly.
10. Maintain the mixture on a heating block at 70°C.
11. Line the heating block with tissue paper, and place multiple square coverslips (18 mm or 22 mm) of #0 or #1 thickness, each coverslip placed approximately 2 inches apart.
12. Using a paintbrush, mark a square outline of wax around the drilled hole on the outer surface of the dish bottom (Methods video S1).
13. Carefully place the petri-dish on the heating block, on top of the coverslip, such that the hole remains at the centre of the coverslip (Methods video S1).
14. Press the cover glass onto the dish very gently with a piece of tissue paper, such that the excess wax flows out and drains on the tissue paper (Methods video S1).

***Note:*** Try to optimize the amount of wax applied to the dish. Too little wax will cause the imaging media to leak, while too much wax might make the wax enter the central cavity region. It should be avoided, as it will interfere with the imaging.

15. When the wax solidifies after a few minutes (the colour will change from transparent to translucent), remove the dish from the block and place it in a box (Methods video S1).
16. Repeat the above steps to prepare multiple such dishes together.
17. Wash the inner surface of the dishes and their lids with distilled water using a squirt bottle.
18. Place the dishes and their lids in a cell culture hood, with the ultraviolet (UV) light and airflow on. Leave the hood slightly ajar to prevent the build-up of air pressure. Leave the dishes overnight to sterilize.
19. The following day, dishes can be removed from the hood and stored in a box at approximately 23°C for long-term usage.

**Pause point:** The dishes can be stored and used over several months. The rest of the preparation is to be carried out just before imaging, as detailed below.

Methods video S1. Preparation of glass-bottom Petri plate, related to steps 8–14 of the before-you-begin section

## Key resources table

**Table:** 

REAGENT or RESOURCE	SOURCE	IDENTIFIER
**Chemicals, peptides, and recombinant proteins**		

Poly-L-Lysine	Sigma-Aldrich	D8414
Na_2_HPO_4_	Sigma-Aldrich	S5136
KH_2_PO_4_	Sigma-Aldrich	P5655
NaCl	Sigma-Aldrich	S9888
Rockout	Calbiochem	555553
SMIFH2	Calbiochem	344092
Latrunculin A	Sigma-Aldrich	L5163
Latrunculin B	Sigma-Aldrich	L5288
Cytochalasin D	Sigma-Aldrich	C8273
Jasplakinolide	Sigma-Aldrich	J4580
Colchicine	Sigma-Aldrich	C9754
Taxol	Sigma-Aldrich	PHL89806
CK-666	Sigma-Aldrich	SML0006
Phalloidin-TRITC	Sigma-Aldrich	P1951
Hoechst 33342	Sigma-Aldrich	14533
DMSO	Sigma-Aldrich	D4818

**Experimental models: Organisms/strains**		

*Canton S*	Bloomington Drosophila Stock Center	BL_64349
*Oregon R-P2*	Bloomington Drosophila Stock Center	BL_2376
*sqh:utr-GFP*	Thomas Lecuit, CNRS France	NA
*protB-dsRed*	John Belote, Syracuse University, USA	NA
*sqh::sqh-GFP*	Bloomington Drosophila Stock Center	BL_57145
*SG18.1Gal4*	Bloomington Drosophila Stock Center	BL_6405
*dj-GFP*	John Belote, Syracuse University, USA	NA
*E-Cadherin-GFP*	Bloomington Drosophila Stock Centre, USA	BL_60584
*UAS-GFP*	Bloomington Drosophila Stock Center	BL_5431

**Software and algorithms**		

Fiji	https://imagej.net/software/fiji/	Open-source

**Other**		

35 mm petri-dish	Falcon	353001
Coverslips (square 18 mm or 22 mm)	Blue Star, India	NA
Parafilm-M	Bemis Company, Inc, USA	NA
Whatmann^TM^ filter paper	Sigma-Aldrich	Grade 1
Forceps	Fine Science Tools	Dumont #55 and #3
Cavity Slide	BLUE STAR micro slides & cover glasses, India	One concavity microslide
Confocal Microscope	Olympus Co. Japan	Olympus FLUOVIEW FV1000 and FV3000 confocal microscope systems mounted on IX71 and IXPro series microscopes, respectively.
Wide field epifluorescence microscope	Nikon Instruments Co. Japan	Eclipse Ti2 Automated inverted microscope
Camera	Oxford Instruments, UK	Andor iXon897 EMCCD
Software	Nikon Instruments Co. Japan	NIS elements
Stereozoom microscope	Leica Microsystems GmBH, Germany	Model M80

## Materials and equipment

The materials used for this protocol are listed in the Key Resource Table. Phosphate Buffered Saline (PBS) was prepared according to the recipe listed in the book ‘Molecular Cloning: A laboratory manual’ by Sambrook and Russell (2001). (http://cshprotocols.cshlp.org/content/2006/1/pdb.rec8247)

The essential equipment used for these experiments are Olympus FLUOVIEW FV1000 and FV3000 confocal microscope systems mounted on IX71 and IXPro series microscopes, respectively. All confocal images were collected using InGAsP-HSD photomultiplier-tube (PMT) detectors. We used a Nikon Eclipse Ti2 inverted fluorescence microscope fitted with an Andor iXon 897 EMCCD camera for wide-field imaging that was captured using the NIS elements software.

## Step-by-step method details

### Preparation of petri-dish just before imaging

**Timing: 25–30 min**

The following procedure of preparation of the petri dish is to be done just before live imaging. It involves making the coverslip surface adhesive so that the isolated testes can stick on it, which is essential for long-term time-lapse imaging with minimal movement of the testis.

1. Gently wipe the surface of the coverslip with lint-free tissue paper, and place the dish on clean tissue paper.
2. Add 50–80 μL poly-L-Lysine in the cavity, and leave undisturbed for 15–20 min.

**CRITICAL:** During the Poly-L-Lysine incubation period (Step 2), set the imaging parameters and dissect the testes (Methods video S2) as detailed in the following sections. Once the testes are dissected and the incubation period is over, proceed with step 3.

3. Remove the poly-L-Lysine using a micropipette and give three quick washes using approximately 50 μL 1**×** PBS per wash to remove residual poly-L-Lysine, as it may affect the viability of the testis.
4. Add fresh 1**×** PBS (80–100 μL) in the cavity, which will serve as the live imaging medium. Alternatively, if applicable, add the working solution containing the drug instead.

**CRITICAL:** The working solutions of the drugs are to be prepared in 1**×** PBS. We found significant effects of DMSO on sperm release and actin cap dynamics at more than 0.8% concentrations. Therefore, one must take care to dilute the stock solutions by at least 100-folds for preparing the working solution. Always use freshly prepared working solutions.

The effective inhibitory concentrations used are: Rockout - 200 μM; SMIFH2- 25 μM; Latrunculin A- 5 μM , Latrunculin B- 10 μM; Cytochalasin D - 2.5 μM and 20 μM; Jasplakinolide - 50 μM; Colchicine - 10 μM; Taxol - 10 μM; and CK-666 - 250 μM.

Methods video S2. Testis dissection from adult Drosophila, related to step 9 of the main protocol

### Setting the imaging parameters on the confocal microscope

**Timing: 5 min**

This section describes the imaging settings on the confocal microscope. The system should be switched on and parameters set before the sample is prepared to begin imaging immediately after preparing the samples.

5. Set the light path for the relevant fluorophores according to the excitation and emission bands, set the frame size to 512**×**512 pixels, and make imaging mode simultaneous to increase the time-resolution, unless there is bleed-through between the specific fluorophores used. For example, the typical setting for the GFP would be – laser line 488 nm for excitation, dichroic FT 500 nm, and emission band 500–520. For combined imaging with GFP and dsRed labels, one could use lasers 488 nm and 560 nm for simultaneous excitation, a double dichroic FT 500/20 and 570/10 with the emission bands 500–520 nm and 570–590 nm.
6. Depending on the type of experiment, set the required modalities- For example, for Multi-Area Time-Lapse imaging (MATL), initialize the stage; for laser ablation or Fluorescence-Recovery-After-Photobleaching (FRAP), activate the relevant modules.

### Dissection of *Drosophila* testis

**Timing: 5 min**

The following steps describe methods for testis dissection and placement of the dissected tissue onto the dish.

7. Fill the dissection cavity with 1**×** PBS.
8. Anesthetize *Drosophila* males of relevant genotype using carbon dioxide or on a cold plate and transfer them onto the dissection cavity, using forceps. Do not pinch the posterior half of the body during this step. Place the dissection cavity under a stereoscope.

***Note:*** Since the testes are nearly translucent, placing a piece of black paper under the dissection cavity and adjusting the light source orthogonal to the viewing angle helps to see them with good contrast.

**CRITICAL:** Make sure to wipe the forceps with lint-free tissue paper soaked with distilled water before and after every experiment to wipe off any residues or drug solutions.

9. Dissect out the testes from each fly as shown in the Methods video S2
  a. Snip the abdomen using a pair of fine forceps (Dumont #55), as shown in the Methods video S2.
  b. Disengage the pair of testes from the gut and cuticle as shown in Methods video S2. Remove the rest of the body and wipe off on tissue paper.
  c. Gently remove the accessory glands and separate the testes from one another by pinching the connection between them.

**CRITICAL:** Care must be taken not to damage the testis or pinch it during the dissection, as demonstrated in Methods video S2.

### Placement of testis on live imaging dish

**Timing: 5–10 min**

10. Gently pick up the dissected testes using a pair of forceps and place them on the surface of the coverslip of the petri dish containing the imaging medium (1**×** PBS or 1**×** PBS+drug, as applicable) (Methods video S3).
11. Using the forceps, gently adjust the inherent coiled shape of the testes, and ensure that it sticks to the poly-L-lysine coated surface. To monitor the coiling stages, actin cap dynamics or the sperm release, the coiled base of the testis should be placed flat against the surface of the coverslip. Make sure the seminal vesicle does not come in between the testicular base and the coverslip (Methods video S3).

**CRITICAL:** Do not forcibly push the testis down or stretch the base against the coverslip surface to flatten it. This can perturb the sperm bundle organization and affect actin dynamics and sperm release events.

12. Place multiple testes in a similar manner close to each other.
13. Soak a sheet of Whatman filter paper with PBS and place it at the side of the dish to maintain humidity in the chamber (Methods video S4).
14. Seal the dish using Parafilm™ and place it on the microscope stage. In the case of drug treatments, make sure to start imaging immediately (Methods video S4).

Methods video S3. Mounting dissected testis on Poly-L-lysine coated coverslip, related to steps 10–12 of the main protocol

Methods video S4. Preparation of the Petri dish for imaging, related to steps 13 and 14 of the main protocol

### Imaging the sample

**Timing: [variable]**

15. Identify the suitable samples using bright-field or DIC optics, which are appropriately mounted and appear intact, using a 10**×** objective (Figure 1A).
16. Switch to the relevant higher objective according to the experimental requirement.
  a. For documenting individualization and coiling events, use 10**×**, NA 0.5 or higher.
  b. For high-resolution analysis of individualization, use 40**×**, NA 1.3 or higher.
  c. For documenting sperm release events, use 20**×**, NA 0.7 or higher
  d. For actin cap dynamics and laser ablation, use 60**×**, NA 1.4.
  e. For Fluorescence Recovery After Photobleaching (FRAP), use 100**×**, NA 1.4.
17. Using the wide-field epi-illumination for a quick scan of the specimen before starting the confocal imaging and locate the general region of interest (ROI) position within the testis. For example, to study actin cap dynamics or nuclei bundle (NB) morphogenesis, identify an NB that is closest to the objective and placed flat along the imaging plane as shown in Figures 1B and 1C.
18. Switch to the confocal mode of imaging and set the rest of the imaging parameters as detailed below
  a. Set digital zoom at 3**×** for all high magnification imaging, and as appropriate for low magnification imaging, to fully capture the relevant ROI. Please note that the NBs move considerably during long-term imaging; hence, set the frame size accordingly to accommodate a sufficiently wide area to catch the entire morphogenesis. It can be achieved by applying an appropriate digital zoom or by changing the magnification of the objective.
  b. Set the z-slice thickness at 0.5 Nyquist and the depth (number of z-steps) with a sufficient buffer of extra digital slices to capture the moving NBs. This judgement is especially critical for high-magnification imaging as NB can move and change orientation.
  c. Set the scan speed to 4 μ seconds/pixel or less, and adjust the frame iterations/time-resolution as per the experimental requirement. For example, to monitor the actin dynamics of the caplets, one would require a shorter time interval, whereas to monitor the gross morphological changes, such as cyst movement or NB morphogenesis for a period of a few hours, a more significant time gap would help to minimize photodamage (see Table 1 and 2 for details).

**Table 1:** Time-interval for monitoring different types of actin dynamics

Nature of experiment	Time-interval
Gross changes during individualization and coiling; sperm release	5–10 min
Actin cap dynamics	Free-run (∼30 s)
FRAP/ laser ablation	Free-run (∼10 s)

**Table 2:** Microscope settings for photobleaching experiments

Nature of experiment	Microscope/ Objective used	Laser settings	Bleach period	Scan speed during bleaching	Bleach area
FRAP	Olympus FV3000/ 100**×**, NA 1.4	100% laser power (∼2 mW 488 nm)	2 s	2 μs/ pixel	1.4 μm^2^ (10**×**10 pixels at settings used)
Laser ablation	Zeiss 710 (Spectra-Physics Mai Tai Ti-Sapphire laser system)/ 63**×**	60–70% laser power (∼ 400 mW) 800 nm IR laser	5 iterations	12.6 μs/ pixel	0.75 μm^2^ (10**×**10 pixels at settings used)
  d. Maintain the laser power between 20–50 μ Watts, the detector HV to 550–620 and detector Gain between 1 and 1.5, respectively, for confocal imaging.
  e. For wide-field imaging, use an LED-based illumination system or Mercury-Halide illumination with a light guide.

***Note:*** The detector settings are applicable for a 1.4 NA objective. The sperm release and coiling are highly sensitive to light and one should maintain minimum possible laser power and optimize the time interval as indicated in Table 1 below. The conditions for the FRAP and Laser ablation are indicated in Table 2. For experiments that do not require free-run imaging (see Table 1), multiple samples can be imaged together using the multi-position module (MATL) for simultaneous xyzt image acquisition from multiple testis specimens.

## Expected outcomes

### Individualization to coiling

In adult testis, cysts at different stages of maturation can be identified by their relative position within the testis and the morphology of the spermatid head bundle (NB) marked by ProtB-dsRed and associated actin structures marked by *sqh::utr-GFP* (Figure 1A).

**Figure 2:**
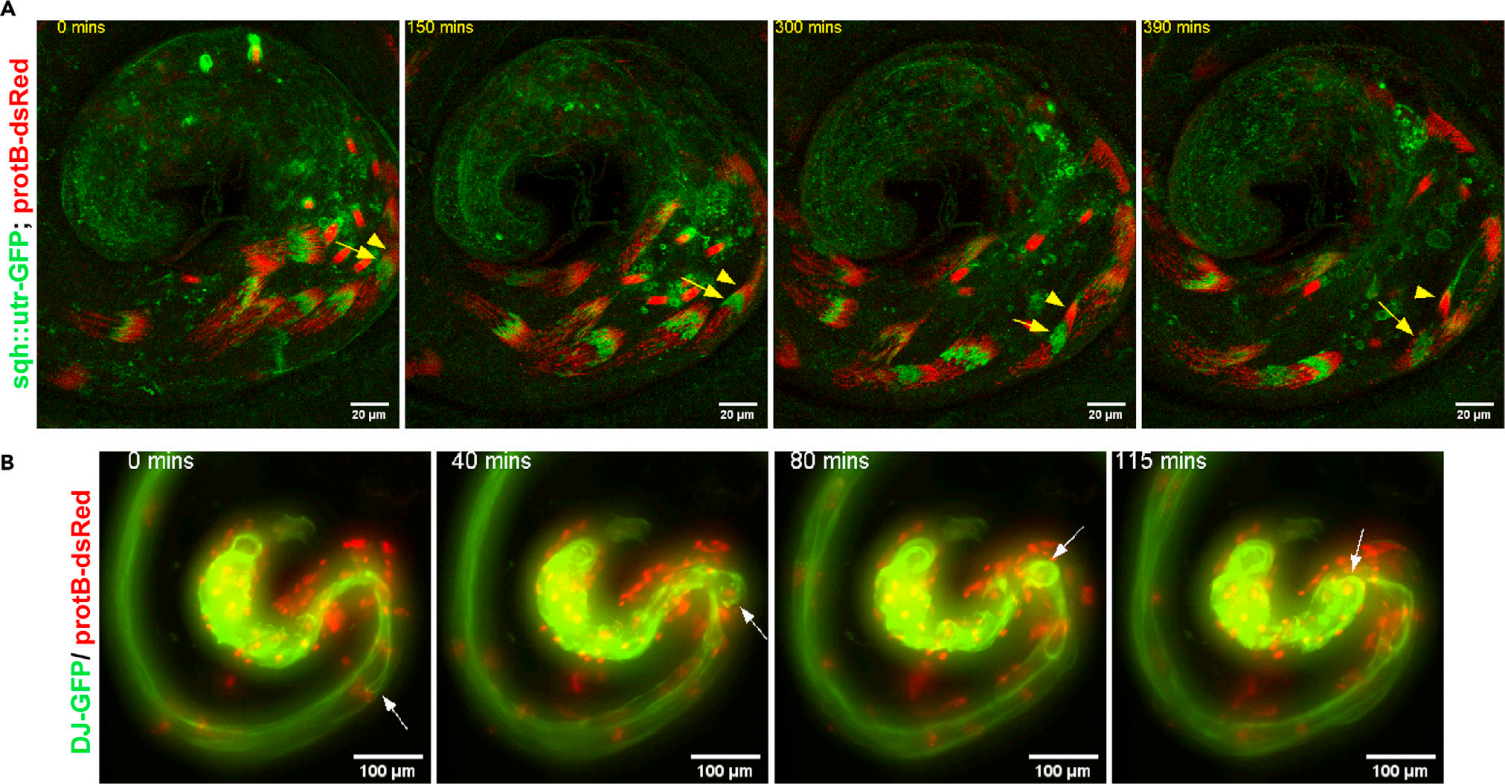
Capturing gross morphological changes during the individualization-to-coiling transition (A) Long-term confocal imaging of *sqh::utr-GFP/+; protB-dsRed/+* testes revealed that the progression of F-actin cones (green, arrow) coincides with the NB compaction (red, arrowhead). Please see Methods video S5A and Kapoor et al. (2021) for more detail. (B) Coiling of spermatid tails (arrow) and the cyst movement into the TE zone in *dj-GFP/ protB-dsRed* testes captured by wide-field microscopy (Methods video S5B). The images were captured with the EMCCD camera fitted on the wide-field epifluorescence microscope using a 20**×** NA 0.9 objective.

During early individualization stages, the NB is conically arranged (arrowhead, Figure 2A), and the early individualization complex is present just caudal to the NB (arrow, Figure 2A; Methods video S5A) (Desai et al., 2009). As the individualization complex proceeds, the NB becomes parallelly arranged, and over time, actin accumulation around the NB could be seen (Desai et al., 2009; Kapoor et al., 2021). During this period, the spermatid tails (identified by *DJ-GFP*) coil (arrow, Figure 2B; Methods video S5B) and the entire cyst assembly moves into the terminal epithelium (TE) zone.

Methods video S5. Time lapse images of individualization and spermatid tail coiling, related to step 18 of the main protocol and the details are provided in Figure 2 legend

### Sperm release

**Figure 3:**
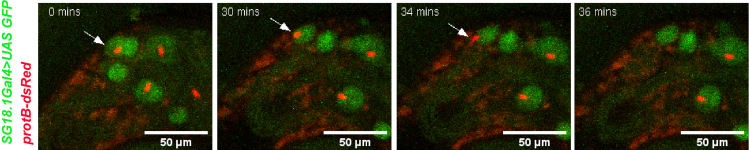
Imaging of spermatid release Release of spermatid occurs by retraction of the head bundle (red) (arrow, 34 min) from the HCC (green) in *SG18.1Gal4>UAS GFP; protB-dsRed* testis. Please see Methods video S6 and Dubey et al. (2016) for more detail.

The cysts enter the TE zone with the NBs facing towards the SV; however, those close to the release site are oriented with the NB facing away from the testicular duct at the base, suggesting that the NB reorient themselves before release (Dubey et al., 2016). Furthermore, spermatids are released tail-first, followed by retraction of the head bundle from the HCC (Dubey et al., 2016) (arrow, Figure 3; Methods video S6).

Methods video S6. Time lapse images of sperm release from an adult Drosophila testis, related to step 18 of the main protocol and as per the description provided in Figures 3 legend

### Imaging of actin cap and performing drug treatments

High magnification imaging of the NB and genetic analyses revealed that the actin cap consists of two domains- WASp dependent caplets (Dubey et al., 2016) and the Dia/DAAM-dependent basket (Kapoor et al., 2021). The forward movement of the spermatid heads into the HCC is repelled by *in situ* assemblies of a caplet, which pushes back the spermatid head, and subsequently disassembles (Dubey et al., 2016), while the basket gathers the sperm heads together, laterally (Kapoor et al., 2021).

**Figure 4:**
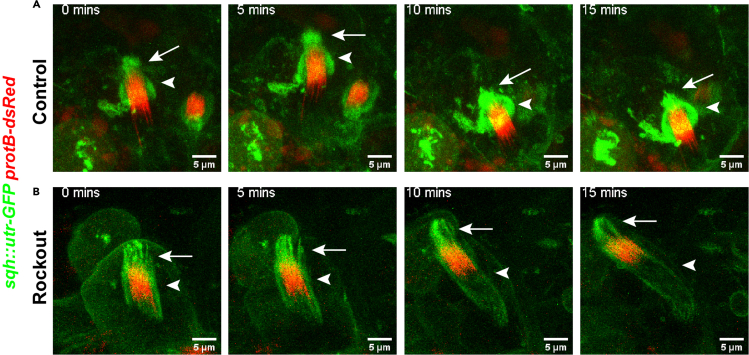
Imaging the actin cap and NB dynamics after the pharmacological treatment of testes High-magnification time-lapse images of NB (red) and actin cap (green) from *sqh::utr-GFP/+; protB-dsRed/+* testes, in the presence of DMSO (A) and 200 μM Rockout (B). Distinct effects on the morphologies of the basket (arrowhead) and caplets (arrow) could be seen upon the Rockout treatment. Please see Methods video S7 and Kapoor et al. (2021) for more detail.

Drug treatments can be used to understand the roles of various cytoskeletal elements during spermatid maturation and release in a time-correlated manner. The testis can be mounted in drug solutions instead of 1**×** PBS as the imaging medium and just prior to the start of imaging. For example, treatment of the testis with the ROCK inhibitor, Rockout, led to sheering the basket and NB integrity, suggesting that the basket structure depends on the kinase Rok and myosin II motor (Kapoor et al., 2021) (Figure 4; Methods video S7). Various other drug treatments can be performed using the drugs and standardized concentrations as listed in the Key Resource Table. For details of the observed phenotypes, refer to Dubey et al. (2016) and Kapoor et al. (2021).

Methods video S7. Time lapse images of actin cap dynamics in wild-type and Rockout treated testes, related to step 18 of the main protocol and as per the description provided in the Figures 4 legend

### Dynamics of actin in the actin cap

**Figure 5:**
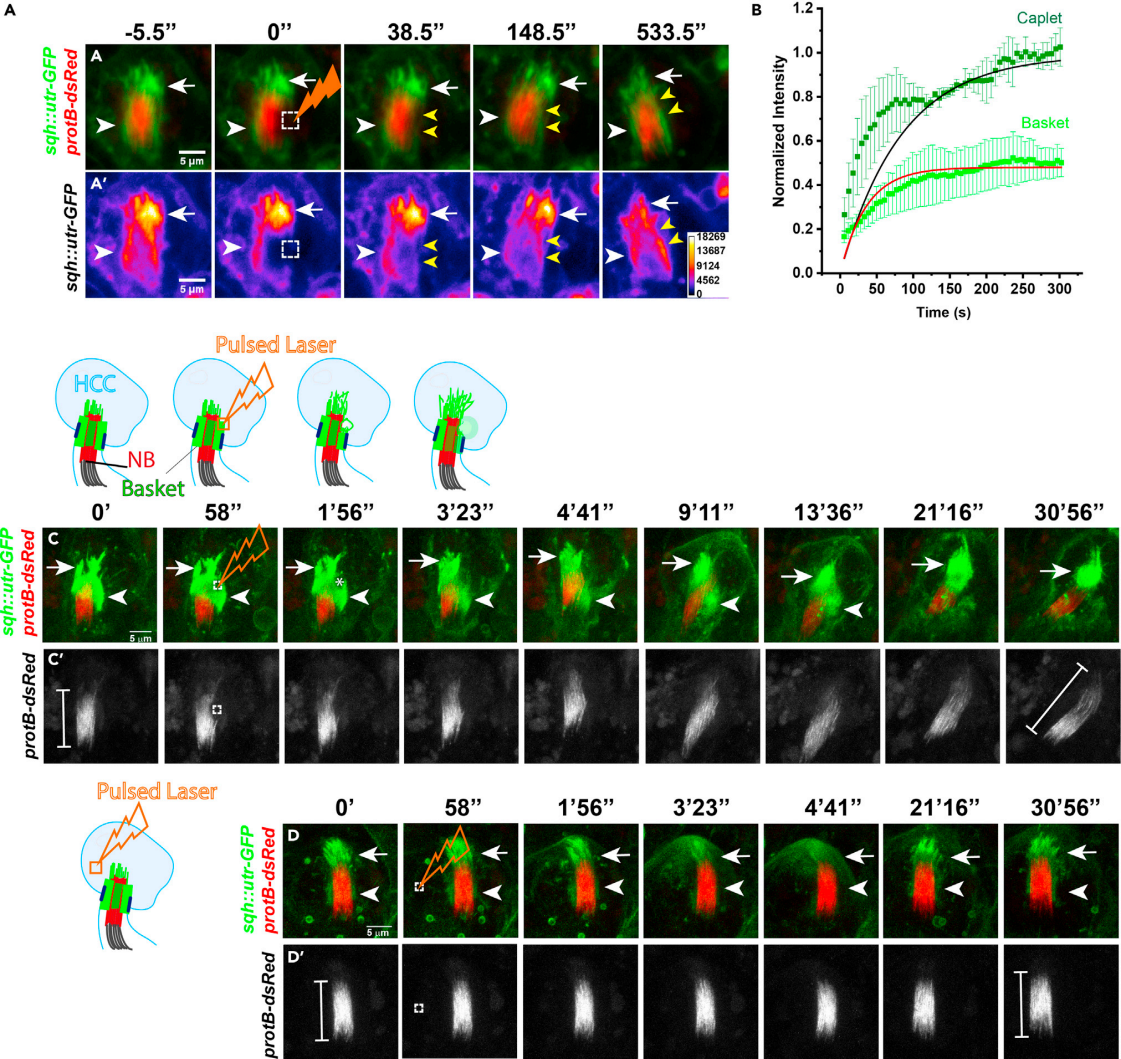
Imaging F-actin dynamics in the basket region of an actin cap (A and B) FRAP (Fluorescence Recovery After Photobleaching) of the basket (A and A′) and caplet domains from *sqh::utr-GFP/+; protB-dsRed/+* testes reveals that caplet recovers faster than basket (B). The error bars indicate +/- S. D. (n = 3) and the plot was fitted with the Levenberg-Marquardt iteration algorithm. (C and D) Laser ablation of the basket (dashed square, C–C’’) leads to sheering of the basket and NB (C) over time, whereas ablating a cytoplasmic region of the HCC (D and D′) does not lead to a similar effect. These results and figures are reproduced from a previous publication (Kapoor et al., 2021) as per the CC BY 4.0 license.

The dynamics and role of these actin structures can also be measured by laser ablation and FRAP studies. The nature of their actin dynamics also revealed the differential properties and functions of the caplet and basket. Bleaching a region of the caplet and basket revealed different recovery profiles for the Utr::GFP, highlighting the difference in the nature of actin dynamics and the roles of the actin substructures in these two domains (Figures 5A and 5B; Kapoor et al., 2021). FRAP was performed on the basket region as per the imaging parameters listed in Table 2. The FRAP recovery profile revealed that caplet actin recovers faster than basket (Figure 5C), which is in line with the rapid and reactive nature of the caplet assembly and disassembly, as opposed to the basket, which forms slowly over time (Dubey et al., 2016; Kapoor et al., 2021).

The role of the basket was further highlighted by cutting a region of the basket (using imaging parameters as described in Table 2) using a high-intensity IR laser. It led to retraction of actin in the ablated zone and subsequent sheering of the basket and NB, as indicated (Figures 5C–5C’’). No such effect was seen upon ablation of a control spot in the HCC (Figures 5D and 5D′). These observations revealed that the basket actively maintains NB bundling through myosin activity, which is disrupted due to the Rockout treatment (Kapoor et al., 2021).

## Limitations

So far, we have successfully used this technique for monitoring short-term dynamic changes in the caplet (Dubey et al., 2016) and basket (Kapoor et al., 2021) domains and long-term morphological changes such as sperm release (Dubey et al., 2016) and NB morphogenesis (Kapoor et al., 2021) for approximately 4–6 h. Successful imaging for more extended periods would likely require the use of fluorophores with better quantum yields and a certain degree of nutrient supplementation to keep the tissue alive for relatively long durations.

Furthermore, due to the thickness of the tissue, it is only possible to image half the depth of the testis at high magnification. The confocality of the image data reduces proportionately with imaging depth, i.e., the image plane being further away from the coverslip surface. Typically, an adult testis is ∼100 μm thick at the base, whereas the confocality is restricted to 50 μm from the coverslip surface. Although this is sufficient to monitor the dynamics and extract the necessary information and quantifications, there can be a loss of data if the NB of interest moves out of the confocality zone during imaging. This constraint may be mitigated by imaging on a two-photon microscope, although it has not been attempted so far.

## Troubleshooting

### Problem 1

Testis does not stick to the coverslip (Step 11).

### Potential solution

Increase the incubation period of the poly-L-Lysine (Step 2), and make sure to not over-wash the dish after removing the Poly-L-Lysine (Step 3). Alternatively, expose the dish to 125 mJ UV (320 nm) crosslinking dose after applying the Poly-L-Lysine mixture, or coat the slides with 10 μg/mL Coring® Cel-Tak according to the manufacturer’s guidelines.

### Problem 2

The testis is punctured during dissection (Step 9) or mounting (Steps 11–12).

### Potential solution

Use a combination of sharp (Dumont #55) and not-so-sharp (e.g., Dumont #3) forceps. Use the sharp one to dissect and discard unnecessary tissue material, and use the blunt one to transfer the testis into the petri-dish. While transferring the testis from the dissection cavity, also pick a residual amount of PBS, along with the testis, using the forceps.

### Problem 3

Difficulty in pushing the sample down to the surface of the coverslip (Step 11).

### Potential solution

Before mounting the sample, add a minimal amount of PBS in the petri-dish, just enough to spread onto the glass bottom area of the dish. After mounting the sample, add the rest of the medium very slowly and carefully. Use an eyelash tip to orient and press the tissue against the coverslip.

### Problem 4

The base of the testis is not in focus while imaging.

### Potential solution

While transferring the sample from the dissection cavity to the coverslip, mount it with the base-first and adjust the rest of the tissue (Methods video S3). Make sure the base region is not resting atop the seminal vesicle.

### Problem 5

The region of interest has moved out of the imaging zone (Step 19).

### Potential solution

This issue is most frequently encountered because of the inherent extensive cyst movement at the testicular base. Adjust the zoom/ size of the imaging area and z-thickness to reduce sample loss. Also, mount multiple testis preparations in the same dish.

## Resource availability

### Lead contact

Further information and requests for resources and reagents should be directed to and will be fulfilled by the lead contact, [Krishanu Ray] (krishanu@tifr.res.in).

### Materials availability

This study did not generate new unique reagents.

## Data Availability

This study did not generate/analyze [datasets/code].
